# Preparation of Selective and Reproducible SERS Sensors of Hg^2+^ Ions via a Sunlight-Induced Thiol–Yne Reaction on Gold Gratings

**DOI:** 10.3390/s19092110

**Published:** 2019-05-07

**Authors:** Olga Guselnikova, Vaclav Svorcik, Oleksiy Lyutakov, Mohamed M. Chehimi, Pavel S. Postnikov

**Affiliations:** 1 Department of Solid State Engineering, University of Chemistry and Technology, 16628 Prague, Czech Republic; Olga.Guselnikova@vscht.cz (O.G.); vaclav.svorcik@vscht.cz (V.S.); oleksiy.lyutakov@vscht.cz (O.L.); 2 Research School of Chemistry and Applied Biomedical Sciences, Tomsk Polytechnic University, Tomsk 634050, Russian; 3 ICMPE (UMR 7182), CNRS, UPEC, Université Paris Est, F-94320 Thiais, France

**Keywords:** SERS, surface modification, Hg^2+^ sensors, water analysis

## Abstract

In this contribution, we propose a novel functional surface-enhanced Raman spectroscopy (SERS) platform for the detection of one of the most hazardous heavy metal ions, Hg^2+^. The design of the proposed sensor is based on the combination of surface plasmon-polariton (SPP) supporting gold grating with the high homogeneity of the response and enhancement and mercaptosuccinic acid (MSA) based specific recognition layer. For the first time, diazonium grafted 4-ethynylphenyl groups have undergone the sunlight-induced thiol–yne reaction with MSA in the presence of Eosine Y. The developed SERS platform provides an extremely sensitive, selective, and convenient analytical procedure to detect mercury ions with limit of detection (LOD) as low as 10^−10^ M (0.027 µg/L) with excellent selectivity over other metals. The developed SERS sensor is compatible with a portable SERS spectrophotometer and does not require the expensive equipment for statistical methods of analysis.

## 1. Introduction

Mercury ions are considered to be one of the most dangerous pollutants in food, water, and the biosphere [1,2]. The most dangerous effect of mercury exposure includes autism and neurodegenerative diseases, such as dementia and Alzheimer’s disease [3,4,5,6]. The high toxicity of mercury ions and their wide distribution in the environment require a design of express and reliable sensors to provide a real-time determination of trace concentrations of Hg^2+^. In recent years, traditional methods for mercury ion detection have become atomic absorption spectroscopy (AAS) [7], inductively coupled plasma mass spectrometry (ICP-MS) [8], atomic fluorescence spectrometry (AFS) [9], gas or liquid chromatography-mass spectrometry (GC-MS) [10], resonance scattering (RSS) [11], and colorimetric methods [12]. To date, considerable progress has been made in the detection of mercury ions. However, current analytical methods are often limited by sensitivity (colorimetric detection), high cost and complex procedures including pretreatment of samples (AAS, AFS, GC-MS), as well as poor selectivity (RRS) [13,14]. Therefore, there is a strong need for the design of a highly sensitive, selective, and convenient analytical procedure to detect mercury ions. In comparison with the above listed methods, surface-enhanced Raman spectroscopy (SERS) has attracted attention for remote and fast monitoring of metal ions [15,16], pesticides and other pollutants [17], explosives [18], and others such as dyes [19] due to its extremely high sensitivity, selectivity, short time of analysis, and nondestructive nature [20,21]; hence the growing popularity of SERS detection in analytical chemistry. 

The majority of SERS sensors for Hg^2+^ detection are based on the assembly of noble metal nanoparticles with a recognition layer or special shapes [13,22,23,24,25], which provide perfect selective recognition. Currently, considerable efforts have been made in order to enhance the sensitivity rather than to achieve reproducibility and reliability of SERS response, which facilitates the implementation and application of this technique in the laboratory and real conditions [26]. However, nanoparticles (NPs) aggregation control and formation of nucleation centers lead to poor reproducibility of SERS signal and restrict the wide applicability of SERS methods [22,27]. Therefore, one of the most prominent trends became a preparation of the high surface area, uniform, inexpensive, easy-to-prepare, sensitive, and selective substrates with a homogeneous distribution of electric field by the preparation of structures with an ordered distribution of NPs or deposition of a thin metal layer on the pre-structured surface. We proposed novel highly-ordered active periodic gold grating, created by excimer laser writing in a short time and on a large scale, and able to support plasmon-polariton as a platform for the SERS sensor system [15,28]. Such design possesses a range of advantages, especially in terms of high homogeneity of response and high enhancement factor, as an increase in the electric field intensity occurs not only at hot spots but homogenously "spreads" over the entire active surface of the sample [15,28,29].

Recently we reported the simple and reliable SERS sensor based on periodic gold grating for the detection of heavy metal ions [15]. The combination of optimal SERS-response with a specific recognition layer—which can be formed via diazonium chemistry—allowed us to determine heavy metal ions with a limit of detection (LOD) of 10^−14^ M. Nevertheless, the design of highly selective sensors towards mercury ions is still in great demand [1,2,3,4,5,6]. Obviously, this task can be solved using an appropriate recognition layer with high affinity to the mercury ions. 

Thiol-containing ligands have been known as perfect capping agents for mercury ions, that anticipate its application for the sensing [30,31]. Several research groups demonstrated different strategies of Hg^2+^ detection by the utilization of L-cysteine [32], cysteamine [33], mercaptosuccinic acid [34], dimercaptosuccinic acid [35], and mercaptopropionic acid [36]. Mercaptosuccinic acid (MSA), being a cheap and commercially available ligand, tending to form a strong complex with Hg^2+^ ions, is one of the best candidates [36,37]. A wide range of metal surfaces can be easily decorated by thiol-containing ligands via chemisorption or covalent approaches [32,33,34,35,36,37], which are beneficial due to the strength of binding. One elegant way to attach MSA is via a click reaction, such as thiol–ene and thiol–yne reactions [38]. Nowadays, several basic approaches of thiol–ene/thiol–yne activation can be distinguished—thermal, utilization of metal catalytic systems, radical initiators, and UV irradiation [38,39]. While UV radiation has been extensively used for the thiol–yne reaction between aryl and mercaptosilane [40], visible light has also been reported as a stimulus for the photocatalytic thiol–yne coupling [41]. Nevertheless, the utilization of sunlight is more preferable in terms of sustainable chemistry and engineering.

For the design of a novel, reproducible, sensitive, and easy-to-prepare SERS sensor for the detection of Hg^2+^ ions, we utilized surface plasmon-polariton (SPP) supporting gold gratings in combination with diazonium modification by 4-ethynylbenzenediazonium tosylate. Attachment of MSA serving as a recognition layer was realized by the visible-light-induced photocatalytic reaction between surface-bound 4-ethynylphenyl layers and MSA. The sensor surfaces and reference materials were characterized by Raman spectroscopy and X-ray photoelectron spectroscopy (XPS). The performances of the sensors will be discussed in terms of selectivity, sensitivity, and reproducibility. 

## 2. Materials and Methods

### 2.1. Materials

Acetic acid (reagent grade, ≥99%), diethyl ether (≥99.7%), 4-ethynylaniline (97.0%), p-toluenesulfonic acid monohydrate (ACS reagent, ≥98.5%), mercaptosuccinic acid (≥99.0%), cobalt(II) chloride hexahydrate (reagent grade), copper(II) chloride (99%), cadmium chloride hydrate (99.995% trace metals basis), lead(II) chloride (99.999%), mercury(II) chloride (reagent grade, 99%), aluminum nitrate nonahydrate (99.997%), zinc chloride (99.999%), chromium(II) chloride (95%), high-purity water (EMD MILLIPORE), and methanol (≥99.8%) were purchased from Sigma-Aldrich and used without further purification. Gold gratings were prepared according to a published procedure [29], as shown in Figure S1 (Supplementary Information), 4-ethynylbenzenediazonium tosylate (ADT-C≡CH) was prepared according to [42], and the modification procedure was conducted according to [43].

### 2.2. Sunlight Induced a Thiol–Yne Reaction between Ethynyl Groups and Mercaptosuccinic Acid

Modified gold gratings were immersed in 10 ml of 20 mM solution mercaptosuccinic acid with 4 mg of Eosine Y in acetonitrile. Afterwards, samples were placed in a visible range photoreactor (Dr Honle, UVA CUBE 400 model) for 1 h. Afterward, reaction samples were washed by acetonitrile (3 times), methanol (3 times), and acetone (3 times). 

### 2.3. Measurement Techniques

The sunlight-induced thiol–yne reaction was carried out in the visible range photoreactor (Dr Honle, UVA Cube 400 sun simulator).

A K Alpha machine (Thermo, East Grinstead, UK) fitted with monochromated Al Kα X-ray source (hν = 1486.6 eV, 400 µm spot size) was employed to analyze the surface chemical composition. Avantage software was used for spectral acquisition and data processing. A flood gun was used to compensate for the charge build up. Spectral calibration was achieved by setting the Au 4f_7/2_ peak at 84 eV [44,45].

Raman scattering was measured on a portable ProRaman-L spectrometer (Laser power 15 mW) with 785 nm excitation wavelengths. Spectra were measured 30 times, each of them with 3 s accumulation time.

### 2.4. Raman Spectroscopy Investigations—Complexation with Metal Ions 

Typically, MSA-grafted gold gratings were immersed in 15 mL of aqueous solution of metal chlorides (CoCl_2_, CuCl_2_, CdCl_2_, ZnCl_2_, HgCl_2_, PbCl_2_, PbCl_2_, Al(NO_3_)_3_) with different concentrations for 20 min. Then, MSA-grafted gold gratings were washed with water, dried in a desiccator and then immediately analyzed by Raman spectroscopy. Limit of detection was calculated from the Signal-to-Noise approach. According to the IUPAC recommendations, this relation must be at least three and the minimal concentration at which this relation is satisfied can be considered as “limit of detection” (LOD). The standard deviation of background signal (Noise) was calculated from 10 spectra of Au-MSA according to $SD=\sqrt{\frac{\sum \left|x-\bar{x}\right|}{N}}$ relation, (where *x* is the value of C=O signal intensity from grafted MSA at 1633 cm^−1^, $\bar{x}$ is the mean of the intensities, N—number of used spectra) and was found to be SD = 1.14. 

## 3. Results and Discussion

Very recently we reported the application of periodic gold gratings as sensitive and express sensors for determination of heavy metal ions, organic dyes, and marker of diseases using SERS [15,28,46,47]. The gold gratings provide a unique reproducibility combined with a high SERS response, which allows for the achievement of extremely high sensitivity due to its unique structure, as shown in Figure S2. The modification of the gratings’ surface by water-soluble arenediazonium tosylates led to covalent grafting of organic molecules, which can be readily transformed with the formation of recognition moieties [15,28,46,47,48]. In this paper, we used a similar approach to the functionalization by the derivatives of mercaptosuccinic acid for the specific binding of mercury ions.

**Figure 1:**
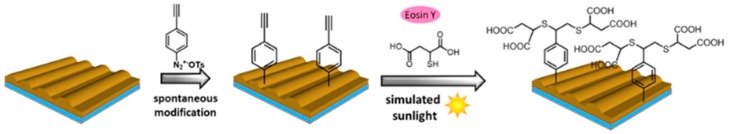
Preparation of gold gratings grafted with mercaptosuccinic acid via thiol–yne reaction under simulated sunlight irradiation.

We started with the spontaneous modification of the gratings’ surface by 4-ethynylbenzenediazonium tosylate (ADT-C≡CH) allowing the formation of the polyphenylene layer with active acetylenic moieties, as shown in Figure 1.

The acetylene group could be easily transformed to corresponding MSA derivatives via convenient thiol–yne click reactions under UV-irradiation directly on the surface [40]. Taking into account the drawbacks of UV to trigger reactions, we proposed the sunlight-induced reaction for the selective modification of ethynyl-moieties directly on the surface.

**Figure 2:**
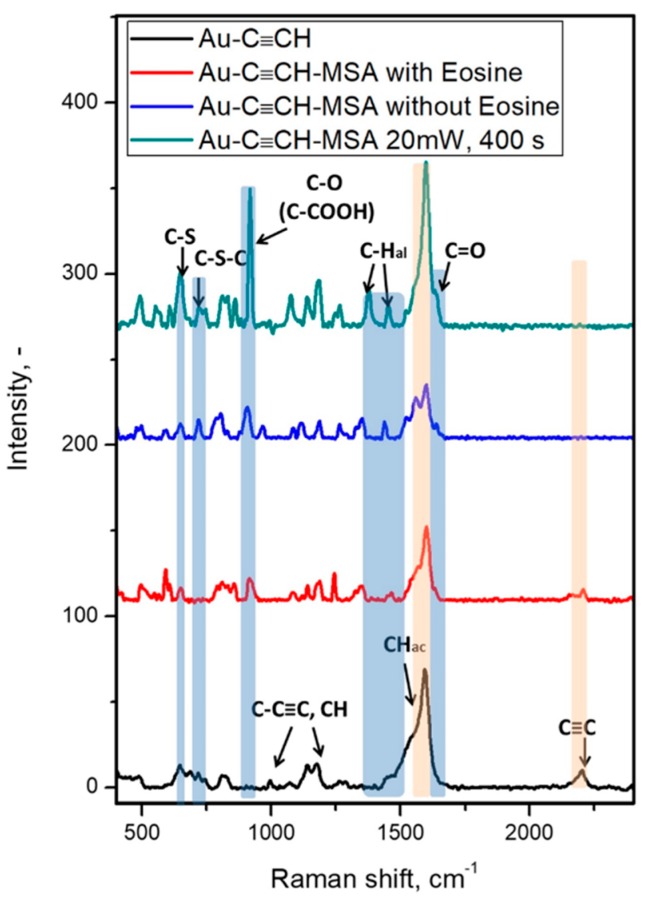
Raman spectra of gold gratings modified by ADT-C≡CH, after grafting with mercaptosuccinic acid (MSA).

Firstly, gold gratings were favorably modified via spontaneous reaction with ADT-C≡CH via simple immersion in 2 mM ADT-C≡CH aqueous solution [43]. Raman spectroscopy enabled the confirmation of successful grafting by the appearance of new peaks related to 4-ethynylbenzene functional groups, as shown in Figure 2: 2198 cm^−1^ (C≡CH stretch), 1594 cm^−1^ (Ar ring stretch), 1174, 1140 cm^−1^ (CH in-plane deformations), 994 cm^−1^ (-C-C≡C stretch), 810, 742 cm^−1^ (CH out of plane deformations), 642 cm^−1^ (-C≡C-H bending vibrations), 492 cm^−1^ (Ar ring vibrations), 400 cm^−1^ (Au-C stretch), 300 cm^−1^ (-C≡CH skeletal vibrations).

**Figure 3:**
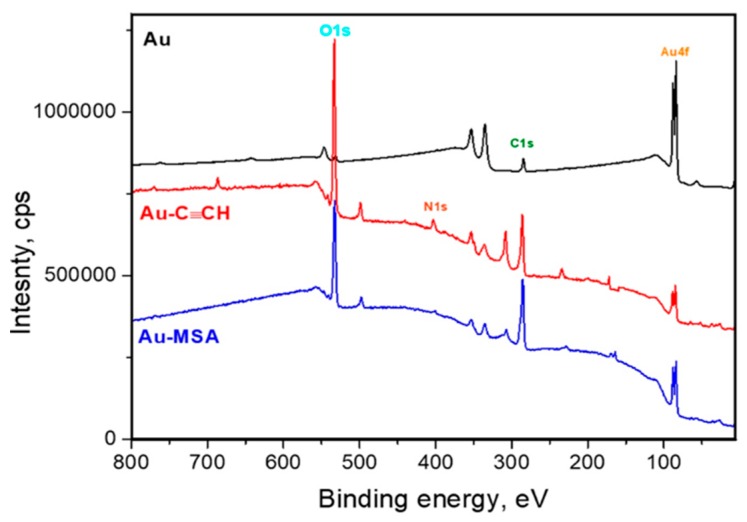
XPS survey spectra from gold gratings grafted with mercaptosuccinic acid via thiol–yne reaction under simulated sunlight irradiation.

Also, XPS permitted the confirmation of the grafting of -C6H4-C≡CH aryl groups to gold, as shown in Figure 3. The covalent modification has resulted in a significant increase of the inelastic background of the gold platform in comparison with the background recorded with bare gold gratings. Attenuation of gold not only induces lower relative peak intensity of gold core level peaks, but also an increase in the inelastically scattered gold photoelectrons, hence the concave-shaped background [40,45]. This is exacerbated after attachment of the second layer (MSA).

It is worth noting that attachment of the aryl layer has induced an increase of the surface carbon content and the C/Au atom ratio, as shown in Table 1. This accounts for the successful procedure of the spontaneous surface modification with aryl diazonium salts, as shown in Table 1.

The initial study of the thiol–yne reaction has been started with simple irradiation by sunlight simulator of gratings, immersed in MSA solution in acetonitrile. The detailed investigations of surface layers revealed the principal possibility of the thiol–yne reaction induced by sunlight. Thus, we observed the appearance of characteristic vibration bands from the carboxy-groups (1634 cm^−1^ (C=O stretch), 1467 cm^−1^ (in-plane OH bending), 1248 and 850 cm^−1^ (OH deformational vibrations), and 520 cm^−1^ (O-C-O), as well as from the C-S bond (652 cm^−1^ (C-S-C stretch) and 593 cm^−1^ (C-S stretch)). The characteristic signals have also been observed in the XPS spectra (C-S S2p, 164 eV), as shown in Figure 2. Unfortunately, we did not reach the full conversion of surface ethynyl-groups according to Raman spectra. After one hour of reaction, we still observed the characteristic band from the C≡C bond at 2198 cm^−1^. Moreover, according to XPS data, we observed a peak relative to oxidized sulfur (SOx S2p, 169 eV), which can be associated with side reactions on the surface, as shown in Figure 3 and Table 1. N at.% decreases upon grafting MSA by the thiol–yne reaction. After attachment, the nitrogen content decreases due to the attenuation of the aryl layer by the MSA moiety. 

In order to achieve the full conversion of surface groups, we implemented the new approach, recently developed by Ananikov, utilizing Eosin Y as a photocatalyst [41]. The simple addition of Eosin Y to the solution of MSA followed by irradiation of gratings in the sunlight-simulator led to the full conversion of surface ethynyl-groups with the formation of appropriate thioethers. After the reaction, Raman spectra of the surface changed dramatically. Firstly, the most character adsorption band of C≡C at 2198 cm^−1^ disappeared, indicating full conversion of the reaction. Moreover, Raman signals at 1634 cm^−1^ (C=O stretch), 1467 cm^−1^ (in-plane OH bending), 1248 and 850 cm^−1^ (OH deformational vibrations), and 520 cm^−1^ (O-C-O) reveal the presence of free carboxyl groups on the surface in the structure of grafted MSA.

Additionally, the appearance of new adsorption bands at 652 cm^−1^ (C-S-C stretch) and 593 cm^−1^ (C-S stretch), and absence of the new peak at 2600 cm^−1^ (S-H stretch) indicate full successful thiol–yne reaction on the surface of gold grating [40]. After the formation of thioethers, one can note the rise of an S2p peak centered at 163.9 eV, as well as an increase in the C1s and O1s relative peak intensities, as shown in Figure 2, due to the clicked MSA. Moreover, the survey spectrum exhibits a substantial increase of the inelastic background, which parallels the attenuation of the Au4f peak from the underlying gold grating [40].

**Figure 4:**
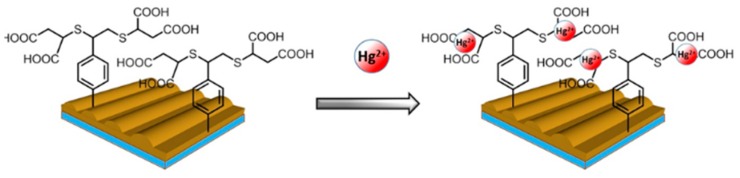
Representation of Hg^2+^ ion capture by gold grating grafted with mercaptosuccinic acid.

The prepared samples with covalently grafted MSA-groups have been tested as a sensor system towards detection of mercury ions in stock solutions with concentrations from 10^−6^ to 10^−10^ M, as shown in Figure 4.

**Figure 5:**
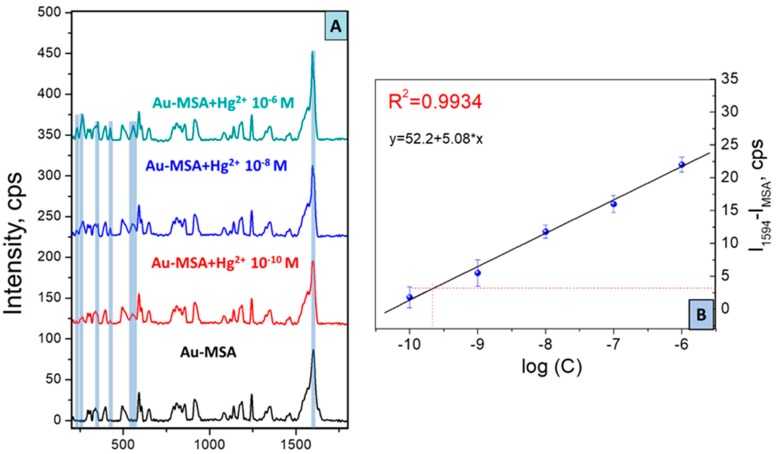
(**A**) Surface-enhanced Raman spectroscopy (SERS) spectra of gold grating grafted with mercaptosuccinic acid in the presence of Hg^2+^ in different concentrations (10^−10^ to 10^−6^ M); (**B**) linear correlation of peak intensity difference (at 1594 cm^−1^ after Hg^2+^ capture and C=O on the Au-MSA) over the concentration range of Hg^2+^ ions.

**Figure 6:**
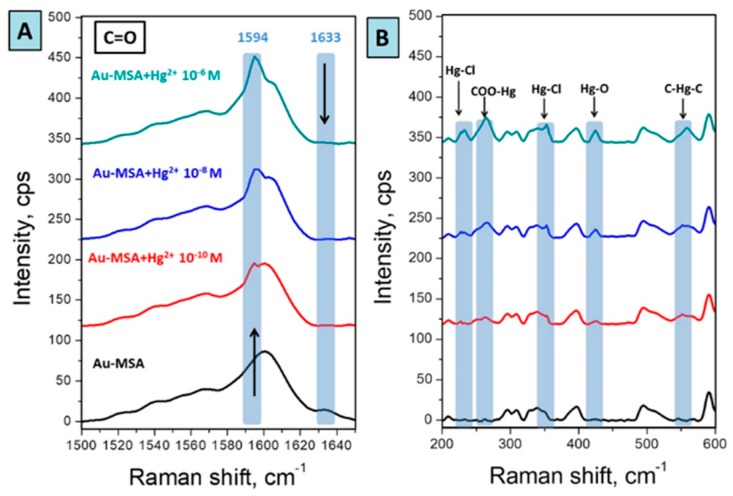
(**A**) Dependence of C=O peak shift in the dependence of Hg^2+^ concentration; (**B**) Enlarged area (200–600 cm^−1^) of SERS spectra from Figure 4, indicating the formation of a complex of MSA with Hg^2+.^

Figure 5 illustrates SERS spectra taken from the Au-MSA after interaction with Hg^2+^ aqueous solutions in different concentrations. SERS spectra underwent the baseline correction and the normalization in comparison with the intensity of the blank sample. After the interaction of MSA functional groups on the surface with Hg^2+^ ions, we observed considerable changes in the area, responsible for the vibrations of the carboxyl group 1580–1650 cm^−1^. The peak at 1633 cm^−1^ (C=O in the structure of MSA) vanished simultaneously with the introduction of a new peak at 1594 cm^−1^, indicating successful capture of Hg^2+^ by the carboxylate group [15,49]. With the increase of Hg^2+^ concentration, the intensity of the absorption band at 1594 cm^−1^ was consistently growing, demonstrating the indisputable formation of MSA-Hg complex, as shown in Figure 6. In addition, the appearance of new peaks at 233, 366 cm^−1^ (Hg-Cl, 265 cm^−1^ (-COO-Hg), 425 cm^−1^ (Hg-O), and 558 cm^−1^ (C-Hg-C) confirm the binding of mercury ions with carboxylate groups [49,50,51].

**Figure 7:**
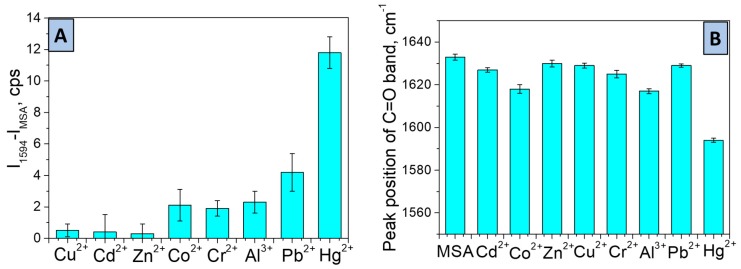
Selectivity tests: (**A**) peak intensity difference for Au-MSA (IMSA) and their metal ion complexes (I_1594_); (**B**) metal-ion induced a frequency shift of the carbonyl group of the SERS sensor. Conditions: initial metal ion concentration = 10^-8^ M.

The standard linear curve was plotted in the coordinates log (Hg^2+^ concentration)—the intensity of peak at 1594 cm^−1^ (attributed to binding state) after subtraction of intensity at 1594 cm^−1^ on Au-MSA sample, as shown in Figure 5. The standard curve of the solution (Hg^2+^) obeys the equation Y = 52.2 + 5.08 × X, which shows a perfect relationship with a linear character and correlation coefficient of 0.9934. According to IUPAC recommendations, the minimally acceptable signal intensity must be three times greater than the background signal deviation, i.e., the intensity of the shifted MSA peak should have an absolute value equal to 3.12. As is depicted in Figure 5, this intensity approximately corresponded to 10^−9.61^ M. The LOD of sensor 10^−9.61^ M is equivalent to 0.049 μg/L; these results imply that quantitative analysis would be feasible in this concentration range. Such a detectable level is much lower than the maximum contaminant level of mercury (2 µg/L) accepted in drinking water and continuous concentrations (0.77 μg/L) of fresh water according to mercury regulation in the United States and Europe [52,53]. The SERS sensor designed so far can thus confidently be employed for water monitoring. Therefore, it can be concluded that the SERS response of the sensor is specific to the Hg^2+^ ion, as shown in Figure 7B. The prepared sensor can compete with world analog sensor systems, taking into account the values of LOD, selectivity, time of analysis, and portability of device [22,23,24,25,26,27,54].

One important feature of sensors is selectivity. Herein, selectivity was demonstrated by the comparison of SERS spectra recorded after interaction of Au-MSA grating with 10^−8^ M aqueous solution of Cu^2+^, Cd^2+^, Zn^2+^, Co^2+^, Cr^2+^, Pb^2+^, and Al^3+^ with targeted Hg^2+^ ions. Figure 7A demonstrates that all metal ions almost do not react with the sensor surface and the corresponding peaks did not significantly shift from the initial Au-MSA peak centered at 1594 cm^−1^.

The negligible increase of intensity was found in the case of Co^2+^, Al^3+^, and Cr^2+^, probably due to physical sorption on the gold surface and more evident increases in the case of Pb^2+^ due to structural similarity to targeted Hg^2+^. As we found before, the position of the Raman signal, responsible for the C=O bond in the structure of chelating functional groups like DTPA can vary after interaction with metal ions due to the formation of complexes. Therefore, we also demonstrated the dependence of peak position of the C=O band on the type of metal ion (Cu^2+^, Cd^2+^, Zn^2+^, Co^2+^, Cr^2+^, Pb^2+^, Al^3+^, Hg^2+^) after interaction with Au-MSA gold grating for 20 min, as shown in Figure 7B. In the case of detection of targeted Hg^2+^ ions, the shift of the C=O peak position was found to be 39, whereas, for all other metals, this value did not exceed 10, except for Co^2+^ and Pb^2+^ (value of shift is 18 and 16, respectively). Therefore, it can be concluded that the SERS response of the sensor is quite specific to the Hg^2+^ ion, as shown in Figure 7B. 

**Figure 8:**
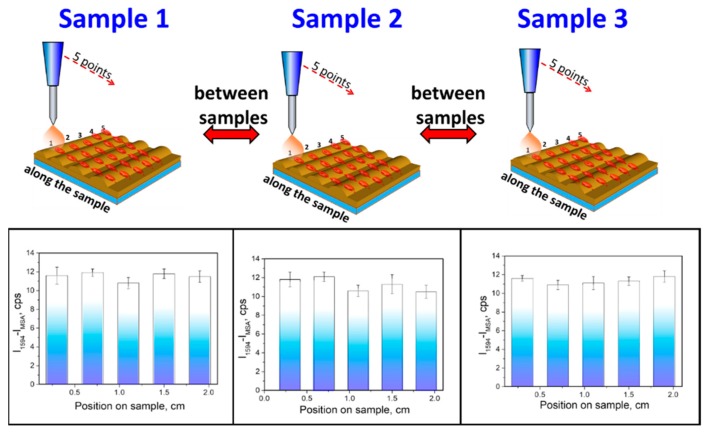
Test of functionalized SERS substrate reproducibility during sensing of Hg^2+^—variation of 1594 cm^−1^ (C=O stretch) peak intensity along the one sample surface (error bars) and between the different samples (column height).

To address the “reproducibility criteria”, one of the most challenging factors in the area of SERS sensor platforms, we tested reproducibility criteria of prepared Au-MSA grating along with the one sample and between three samples, as shown in Figure 8.

For the investigation of reproducibility, we measured SERS spectra on the five lines across the sample, and on every line, five spectra were measured, as shown in Figure 8. The column heights show the mean absolute peak intensities measured on the five lines and the error bars represent the scatter of the values measured at different spots. It was found that the deviation of the peak (1594 cm^−1^) intensity along the sample is 5.2% and the deviation between three samples does not exceed 6.8%. Moreover, SERS measurements were carried out on the portable Raman spectrophotometer with a collection time of spectra of 90 s. By this way, a combination of high homogeneity of the response and high enhancement factor highly-ordered provided by the active periodic gold grating, and two-stage surface modification with MSA enabled the achievement of specific detection of Hg^2+^ and reproducibility even without utilization of statistic methods on the portable Raman spectrometer.

## 4. Conclusions

In this work, we proposed the novel design of a SERS sensor for Hg^2+^ ion detection by the sunlight-induced thiol–yne reaction of 4-ethynylphenyl groups on the surface of SPP supporting gold grating with MSA in the presence of photocatalyst Eosine Y. XPS and Raman results brought strong supporting evidence for the effective grafting of MSA via a sunlight-induced thiol–yne reaction. The Au-MSA gratings were incubated with various metal ions and the corresponding metal-grating complexes monitored with SERS. The Au-MSA SERS sensor has the following figures of merit: specific detection of Hg^2+^ was achieved with LOD as low as 10^−10^ M (0.027 µg/L) and excellent SERS response reproducibility. Indeed, the statistical scatter in the SERS results along the one sample surface does not exceed 5.2% and is less than 6.8% between different samples.

## Figures and Tables

**Table 1:** XPS-deduced surface chemical composition of untreated and modified gold plates as determined.

Materials	Au	C	O	N	S
Au	53.7	34.6	11.7	-	-
Au-C≡CH	14	48.2	32.5	5.3	-
A-MSA	2.5	53.2	39.8	1.3	3.2

## Supplementary Materials

The following are available online at https://www.mdpi.com/1424-8220/19/9/2110/s1, Figure S1: Scheme of SPP supporting gold preparation, Figure S2: AFM and SEM images of gold grating.
