# Supplementary material for: Preparation of Selective and Reproducible SERS Sensors of Hg2+ Ions via a Sunlight-Induced Thiol–Yne Reaction on Gold Gratings
Source: Sensors (Basel). 2019 May 7;19(9):2110. doi: 10.3390/s19092110 (PMC6539914; doi:10.3390/s19092110)
Supplement: Supplementary file 1 [file sensors-19-02110-s001.pdf]

Supplementary information

Preparation of Selective and Reproducible SERS Sensor of Hg2+ Ions via Sunlight Induced Thiol-Yne Reaction on Gold Gratings

Olga Guselnikova[1,2], Vaclav Svorcik[2], Oleksiy Lyutakov[2], Mohamed M. Chehimi[3*] and Pavel S. Postnikov[1,2*]

[1] Department of Solid State Engineering, University of Chemistry and Technology, 16628 Prague, Czech Republic

[2] Research School of Chemistry and Applied Biomedical Sciences, Tomsk Polytechnic University, Russian Federation

[3] Université Paris Est, UMR 7182 CNRS, UPEC, 94320 Thiais, France

Grating preparation Polymer films (Solution of epoxy resin - photoresist, Su-8, purchased from Microchem) were spin-coated (1000 rpm) from a solution onto freshly cleaned glass substrates (supplied by Glassbel Ltd, CR) during 30 min. The prepared samples were dried at 50°C for 24 h and irradiated by UV-source for 30 min. After that, the UV-irradiated samples were dried at 90°C for 2 h. Briefly, the flat polymer surface was patterned by KrF excimer laser (COMPexPro 50F, Coherent, Inc., wavelength 248 nm, pulse duration 20-40 ns, repetition rate 10 Hz). The laser beam was polarized linearly with a cube of a UV-grade fused silica with an active polarization layer. The samples were irradiated with 3500 laser pulses with laser fluencies 9 mJ cm^{-2}. The angle of laser beam incidence with respect to the sample surface normal was 50° and the aperture with the area of 5×10 mm^2 was used. As a result, the periodic surface structures were created on the Su-8 surface with 1x2 cm^2 patterned area size. Then gold was deposited onto a patterned surface by vacuum sputtering (DC Ar plasma, gas purity of 99.995 %, a gas pressure of 4 Pa, a discharge power of 7.5 W, sputtering time 200 s, and thickness approx. 25 nm). The deposition of gold was accomplished from Au target (purity of 99.99 %, provided by Safina, Czech Republic).

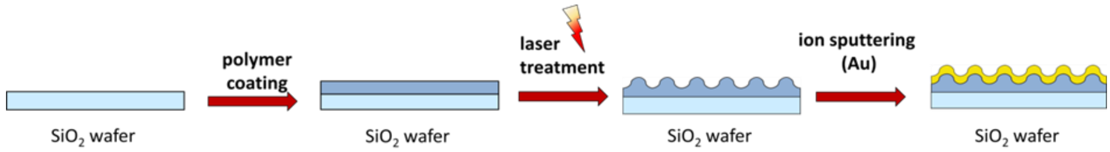

Figure S1. Scheme of SPP supporting gold preparation.

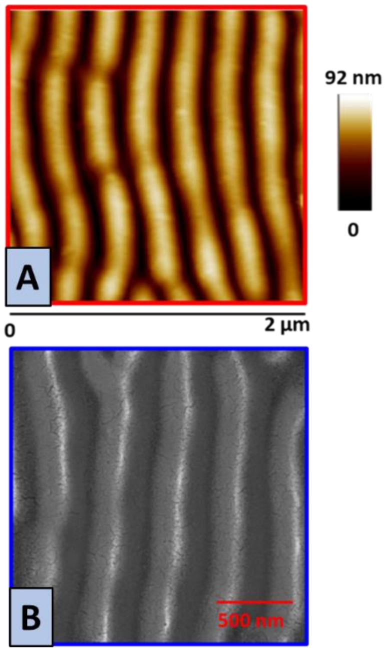

Figure S2. AFM and SEM images of gold grating.
